# Electromagnetic Navigation Bronchoscopy Localization versus Percutaneous CT-Guided Localization for Lung Resection via Video-Assisted Thoracoscopic Surgery: A Propensity-Matched Study

**DOI:** 10.3390/jcm8030379

**Published:** 2019-03-18

**Authors:** Shuenn-Wen Kuo, Ying-Fan Tseng, Kuan-Yu Dai, Yeun-Chung Chang, Ke-Cheng Chen, Jang-Ming Lee

**Affiliations:** 1 Department of Surgery, College of Medicine, National Taiwan University Hospital and National Taiwan University, Taipei 100, Taiwan; shuenn8@gmail.com (S.-W.K.); yohedgehog@gmail.com (Y.-F.T.); dldannylb@gmail.com (K.-Y.D.); ntuhlee@yahoo.com (J.-M.L.); 2 Department of Medical Imaging, College of Medicine, National Taiwan University Hospital and National Taiwan University, Taipei 100, Taiwan; ycc5566@ntu.edu.tw; 3 Institute of Biomedical Engineering, College of Medicine and College of Engineering, National Taiwan University, Taipei 100, Taiwan

**Keywords:** pulmonary nodules, electromagnetic navigation bronchoscopy, dye marking, video-associated thoracic surgery, CT-guided localization

## Abstract

Background: An ideal preoperative localization method is essential for the resection of small and deep-seated pulmonary nodules by video-assisted thoracoscopic surgery (VATS) in the era of low-dose computed tomography (CT) screening. This study describes a new localization method using electromagnetic navigation bronchoscopy (ENB) and compares it against conventional percutaneous CT-guided methods. Methods: Between January 2016 and May 2018, 18 consecutive patients with a total of 27 pulmonary nodules underwent ENB localization using patent blue vital dye before thoracoscopy for lung resection at the National Taiwan University Hospital. Over the same period, 268 patients had a total of 325 pulmonary nodules localized by a CT-guided method. Propensity analysis was applied to minimize bias during comparison. Results: Patients were selected using a propensity-score based process, matched for potential risk factors for localization failure, to ensure equal potential prognostic factors in both groups. After matching, the ENB group had 15 patients with a total of 24 pulmonary nodules, and the CT group had 30 patients with 48 pulmonary nodules. No major procedure-related complications occurred in either group. The target pulmonary nodule was not successfully localized for one patient in the ENB group and three in the CT group. The lesions were fully excised after conversion to mini-thoracotomy. Pathological examination confirmed the accuracy of the dye staining. Analysis found a non-significant difference in the success rate of these two localization methods. However, the following parameters were significantly different: interval between localization to surgery, global time, and rate of pneumothorax (*p* < 0.05). Conclusions: In the era of minimally invasive surgery, surgeons need an efficient one-step way to manage pulmonary nodules. Patent blue vital injection with ENB guidance in the operating room is a new, effective approach to localize small, deep-seated and non-palpable pulmonary lesions, comparable with CT-guided localization.

## 1. Introduction

Identifying pulmonary nodules intra-operatively by direct visualization or palpation can lead to successful resection by video-assisted thoracoscopic surgery (VATS). The increased use of low-dose computed tomography (CT) is revealing greater numbers of sub-centimeter lung lesions. Some of them are deep-seated sub-centimeter pulmonary nodules, which are difficult to palpate or see during VATS, especially those with ground-glass opacity [1]. Nodule detection is dependent on its size and the distance to the pleural surface [2]. Failure to detect a nodule can require conversion to thoracotomy [3]. The conversion rate ranged from 0.7% to 7.5% from the earlier literature [4]. Since lung cancer has emerged as a growing health issue worldwide, including in Asian countries [5,6], VATS for major lung resections has been used in the treatment of lung tumors through minimal invasive surgery, especially for early lung cancer. Low-dose CT has been shown to be effective in the early detection of lung cancer, thus reducing mortality rates [7]. Currently, VATS is often performed through small incision(s), increasing the difficulty of palpation, and thus increasing the difficulty of resecting deep-seated nodules or ground glass opacities. Such cases require preoperative nodule localization for successful intraoperative guidance then followed by lung resection by VATS. CT-guided localization has been shown to be effective preoperatively in recent years; on the other hand, electromagnetic navigation bronchoscopy (ENB) localization of the tumor with dye injection is rapidly emerging to be applied before VATS to achieve the same goal [8,9,10,11,12,13,14,15]. However, no fair comparison of ENB with CT-guided localization has been reported. This study aims to provide a comparison of effectiveness of tumor localization using ENB and CT-guided localization before small pulmonary nodules resection by VATS.

## 2. Materials and Methods

### 2.1. Patients

We reviewed a prospectively maintained database to retrospectively collect patients at National Taiwan University Hospital undergoing localization with CT and ENB followed by VATS from January 2016 to May 2018. Patients were included based on the following eligibility criteria: having lung tumor requiring localization before the operation, deemed medically suitable for surgery, and having undergone thoracoscopy after localization. Preoperative evaluation included medical history review and physical examination, chest CT imaging studies, pulmonary function test, and laboratory examination. Moreover, CT scans of the abdomen, pelvis, brain and bone were performed to identify distant metastasis once suspected. Patients under 18 years old, pregnant, or lactating were excluded. All nodules requiring localization were marked with patent blue vital dye.

The ENB group included patients who had undergone preoperative ENB-guided dye localization for thoracoscopic lung tumor surgery in the operating room at our institute during the study period. The indication for ENB localization was the presence of peripheral small indeterminate lung nodules measuring 0.5–2.0 cm, which were difficult to identify. The Research Ethics Committee of National Taiwan University Hospital approved it. The CT-guided group included patients who had undergone preoperative CT-guided dye localization in the CT room for peripheral small indeterminate lung nodules. The localization procedure was performed by board-certified radiologists on a 16-slice CT scanner (GE LightSpeed; GE Healthcare, Milwaukee, WI, USA) using low-dose exposure and thin slice protocol (1.25 mm thickness, 1.3 pitch, 0.7 s/rotation, 120 kV, 50 mA). These patients were transferred to the general ward after the procedure to await subsequent surgery. All patients were enrolled for propensity score matching. The clinicopathological characteristics of both groups were collected from the patients’ charts.

### 2.2. ENB Dye Localization

**Figure 1:**
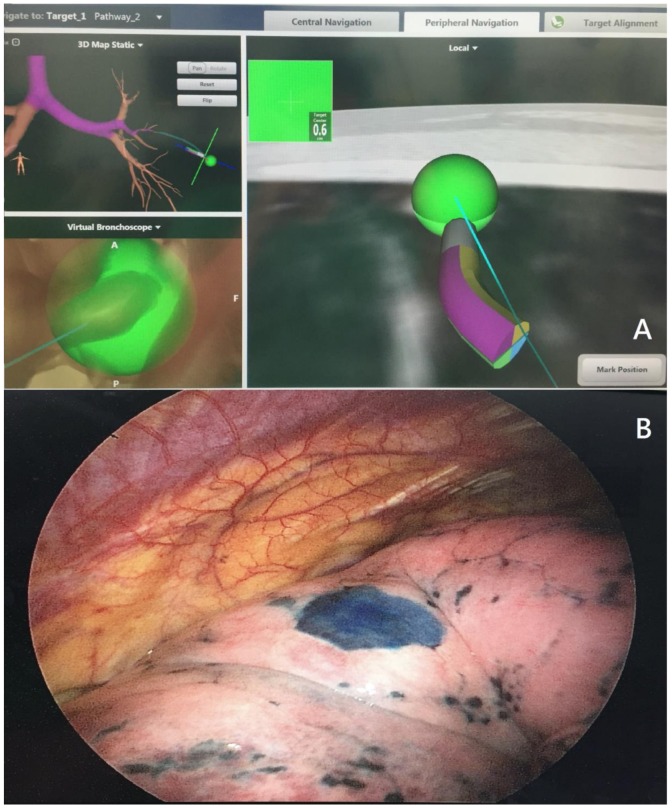
(**A**) ENB navigation for localization of the lung nodule; (**B**) ENB localization shows the lung tumor position by pleural marking during VATS.

Patients underwent general anesthesia with or without intubation before all procedures. We used the computer console of a seventh-edition SuperDimension Navigation System (Covidien, Minneapolis, MN, USA) with planning software to localize and plan a route to the nodules one day prior to the operation. We used the best available exit point from the bronchial tree if no bronchus led to the nodule. Each nodule was localized with a locatable electromagnetic guide, the navigational console and an extended working catheter. The electromagnetic guide was removed after the nodule was located, while the extended working catheter was left in place. We used a bronchoscopic sprayer inserted through the extended working catheter, and injected patent blue vital (0.15 mL) into the nodule. We used an additional injection of no more than 0.15 mL patent blue vital to mark the pleural surface. The patient then was changed to lateral position following by VATS operation (Figure 1A,B).

### 2.3. Percutaneous CT-Guided Localization

**Figure 2:**
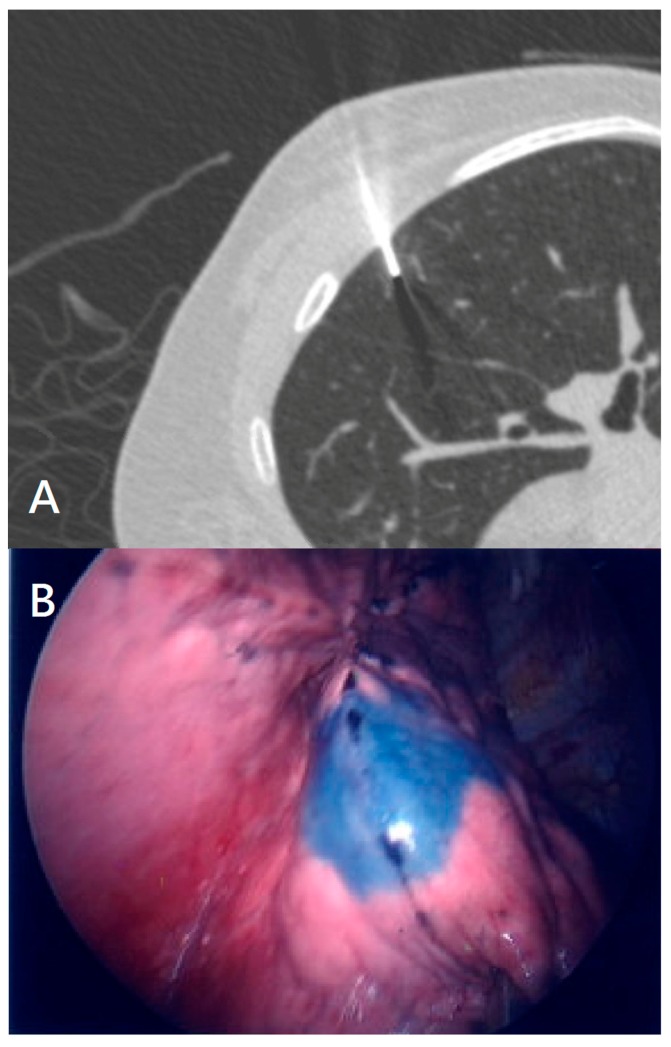
(**A**) CT-guided localization of fine-needle localization with PBV (Patent Blue Vital); (**B**) Pleural marking by CT-guided localization method revealed by VATS.

Patients in this group were sent to the CT room preoperatively on the day of their surgery. The nodule depth was measured as the shortest distance from the nodule to the pleura in the axial view using a standard picture archive and communication system with a commercially available viewer (IMPAX 5.2; Agfa HealthCare, Mortsel, Belgium); it was recorded after consensus was reached. The pathological diagnosis of the resected nodules was classified according to the criteria set by the World Health Organization in 2015. CT-guided PBV (Patent Blue Vital) dye localization has been described in detail elsewhere [14,15]. Briefly, the patient is positioned to provide the shortest access route for needle insertion. In the end-inspiratory breath phase, chest CT was first performed for planning. The needle route was defined by marking the entry and target points of the needle, and then projected on the skin with a laser beam. Local anesthetic (xylocaine 2%; RecipharmMonts, Monts, France) was injected into the skin inlet of the planned localization route in the thoracic wall. Thereafter, approximately 0.2 mL of PBV dye (2.5%; Guerbet, Aulnay-sous-Bois, France) was injected into the deepest part of the nodule via a 22-gauge Chiba needle. Any change in vital signs or significant bleeding caused the procedure to be postponed immediately. Chest CT was performed after the procedure to identify possible complications (including pneumothorax, intrapulmonary focal hemorrhage, or hemothorax) and to evaluate the effect of localization (Figure 2A).

### 2.4. Surgical Procedures

VATS was performed on all patients by attending chest surgeons. Localization with patent blue vital marking and VATS was performed subsequently in the operating room for the ENB group, while percutaneous localization was performed in the CT room for the CT group. The nodule was resected using a linear stapler guided by the dye marking. Every attempt was made to ensure that the nodule was within the specimen (Figure 1B and Figure 2B). After the operation, patients were sent to the ward or intensive care unit for post-operative care. Localization time was defined as the overall time of the procedure. In both groups, surgery time was defined as the time from the first incision to wound closure. Global time was defined as the time from the initiation of the localization to the end of anesthesia (extubation) after surgery in the CT group, and as the time from the induction of anesthesia to the end of anesthesia (extubation) in the ENB group.

### 2.5. Statistical Analyses

The outcome assessed in this study was the success rate of dye localization and lung nodule resection by thoracoscopy. Categorical variables were compared using X^2^ or Fisher’s exact tests, and continuous variables were evaluated using Student’s *t*-test. *p*-Values below 0.05 were regarded as significant. Statistical analyses were performed using SPSS version 18 (SPSS Inc., Chicago, Il, USA), and all statistical tests were two-sided. To minimize bias due to confounding, we estimated propensity scores and matched them using an eighth-to-first digit greedy matching algorithm to create a cohort of matched patients with similar observed characteristics. The propensity score was calculated by logistic regression, which included the operation method, lesion number, depth, size, nodule characteristics with solid component or ground-glass opacity, and patient lung emphysema/COPD (Chronic Obstructive Pulmonary Disease) with pulmonary function test. Patients with similar propensity scores were grouped together. Each patient who underwent localization by ENB was matched with two patients who underwent localization in the CT room. Comparisons of categorical data between both groups were performed with the X^2^ or Fisher exact test. Continuous data were compared using the two-tailed *t*-test. A *p*-value of <0.05 was considered statistically significant.

## 3. Results

### 3.1. Treatments and Outcomes

#### 3.1.1. Patient Demographics and Clinicopathological Characteristics

After propensity score matching with respect to nodule number, size, depth, nodule characteristics with solid component or ground-glass opacity, lung emphysema/COPD, pulmonary function test, and operation method, a total of 45 patients (*n* = 15, ENB-guided group; *n* = 30, CT-guided group) were analyzed. Table 1 summarizes the clinical characteristics of the patients and lung nodules in both groups. There were no significant differences between the groups in terms of age, gender, height, weight, and smoking status, or in terms of nodule number, size, depth, size/depth ratio, GGO (Ground-Glass Opacity)/solid component, pulmonary function test, COPD/emphysema, and location. The final surgical pathology of the lung nodules revealed malignant disease in 87.5% of the nodules in the ENB room group. Of these, 15 and 6 were respectively primary lung adenocarcinoma and metastatic lesions. Three nodules were diagnosed as benign. In the CT room group, malignancies were noted in 89.6% of the nodules. Table 2 provides the detailed pathology results of the resected nodules in both groups.

#### 3.1.2. Localization and Operative Results

Analysis of the matched group for postoperative outcomes demonstrates that the ENB group had significantly shorter global time (143.4 ± 58.0 vs. 258.0 ± 124.0 min, *p* = 0.002), but similar localization time (21.8 ± 12.5 vs. 26.3 ± 14.0 min, *p* = 0.341) and duration of chest tube drainage (1.33 ± 0.8 vs. 1.7 ± 0.8 days, *p* = 0.151). No significant difference was found in surgery time and hospital stay. Neither mortality nor major morbidity were noted in any of the procedures. In both groups, localization-related complications included pneumothorax and intrapulmonary focal hemorrhage. In the ENB group, there was one patient who experienced pneumothorax during the localization procedure (which was relieved after VATS), while the other three patients with focal hemorrhage were observed or received conservative treatment alone. In the CT group, there were 11 patients with pneumothorax, in whom oxygen therapy was given during the waiting time. The incidence rate of pneumothorax between the two groups was significantly different. (*p* = 0.032) Nodule marking was successful by intraoperative identification in 93.3% (14/15) and 90% (27/30) patients in the ENB and CT groups (*p* = 0.711), respectively. All tumors were completely R0-resected (curative resection) by VATS except for one patient with conversion to mini-thoracotomy in the ENB group and three patients in the CT group. Table 3 shows the operative and localization-related information.

## 4. Discussion

Thoracic surgeons are increasingly seeing patients with small lung lesions for possible resection [1,2,4]. Traditionally, open thoracotomy is required for the identification and resection of small lung nodules. However, recent developments in thoracoscopic surgery have led surgeons to favor finger palpation and thoracoscopic visualization to localize nodules, decreasing thoracotomy morbidity [4]. However, several unsuccessful VATS resection cases have shown the limits of palpation during VATS. Transthoracic or bronchoscopic methods using coiling or fiducial markers with CT guidance have improved nodule identification rates, but with some drawbacks [16,17,18,19,20]. Transthoracic CT-guided localization exposes the patient to ionizing radiation [16]. Bronchoscopically placed coils are prone to dislodgment by manipulation of the lung lobe during surgery [16]. This places time constraints on localization and resection because of the diffusion of methylene blue dye or the degradation of radionuclides [17]. The failure rates of transthoracic methylene blue marking and hook-wire localization are respectively reported as 13% and 47%, because of the inability to recognize the dye or due to dislodging of the hook-wire. Moreover, the rate of pneumothorax was 15–30% by transthoracic procedure [12,15,19,20]. However, ENB-guided localization can be achieved intra-operatively on the same table in the same operation room where the surgery can be performed subsequently after localizing procedure, thus preventing patient discomfort due to transportation or waiting, while it is reported to achieve a high success rate of localization [8,9,10,11].

Our 15 matched patients underwent ENB-guided localization with patent blue vital injection as compared to 30 patients with CT-guided localization before VATS lung nodule surgery. Both groups achieved high success rates for localization as well as lung lesion resection with few conversions. There was one localization-related adverse event (6.7%) in ENB group with pneumothorax, while there were 11 cases with pneumothorax in CT group (36.7%). The pneumothorax event correlated with not only the localization methods but also the lung condition of the patients. We did the propensity matching process and equalized the characteristics of the patients. The lung function tests and rates of COPD/emphysema were similar in both groups. Although only conservative treatment was required for them, shortcomings indeed limited the usage in some situations. For example, we did not use CT-guided method for multiple lung nodules (>2 lung nodules) or bilateral lung lesions in fear of localization failure or complications from bilateral or severe pneumothorax. For patients with emphysematous lung or COPD, it is better to choose the ENB-guided localizing method.

Localization techniques for lung nodules are typically reserved for lesions that are small and deep-seated in the lung parenchyma, or lesions that are mainly ground glass opacities. Dye marking is used to enhance localization during surgery and to improve the chances of a successful VATS resection (without conversion to open thoracotomy), but it does not expand the indications for the operation. One benefit of fiducial markers or coils is locating nodules deeper than 2 cm for confident wedge resection that encompasses the fiducial (coil) and the nodule. They provide different information (depth) from dye. Therefore, some advocate double-method localization (dye and coil) [16,17,18]. Our experience demonstrated that ENB-guided transbronchial dye marking before VATS is as safe and efficient as CT-guided localization.

CT-guided localization has the advantage of being reproducible, widely available, and with minor complications if any. On the contrary, navigational tools carries disadvantages of low cost-effectiveness, requiring much labor and experience to master and consuming a lot of resources. Moreover, the CT-guided method can be performed by most chest radiologists and is thought to be more practical. We suggest that there is no single perfect method. It should be dependent on real situations of the institute. Last but not least, ENB has the potential for lung ablation. Bronchoscopic interventional ablation with microwave, RFA, cryotherapy, and photodynamic therapy via the aid of navigational tools was prosperously studied and tested [6,21,22,23,24]. If the ENB can reach the target lesion, clinicians can simply apply the tools to ablate tumors. More evidence should be built to prove this novel concept.

This work is subject to certain limitations. The study is retrospective and is based on the experience of a single medical center, which may incur selection bias. A well-matched control study to compare these methods could minimize such bias. Finally, nodule selection bias exists since some nodules were just followed up due to a perceived difficulty in localization or other nodules were regarded as more easily identified ones and thus were not localized. The matching process helped to minimize the bias. The malignancy rate for the nodules was around 90%, which is considerably higher than in prior studies (48%) [4,8,9,10,11]. However, it is reasonable to suggest that the increased value of the ENB localization outweighs the risk of adverse events, even in patients with benign disease, due to the low number of adverse events. Prospective, multi-institutional randomized controlled studies are required to further demonstrate the superior effectiveness of the proposed approach in lung tumor localization.

Our initial experience with ENB-guided patent blue vital dye marking of small lesions followed by VATS resection suggests that this technique is feasible, safe, and an effective for the resection of lesions that may be difficult to visualize or digitally palpate. Dye marking was successful for the identification of lesions that were small and difficult to palpate, secondary to location or character (i.e., GGOs), under thoracoscopy. Pleural dye was visualized in every dye-marking procedure and, after localization, the extent of resection was guided by careful evaluation of the CT scan in coronal, sagittal, and axial views. In addition, the rarity of complications from the dye localization process and the mean navigation time of 22 min suggest that ENB-guided patent blue vital dye marking is safe and effective.

## 5. Conclusions

ENB with patent blue vital localization intraoperatively identifies small lung nodules as effectively as CT-guided localization in the CT room. The high rate of successful localization, with low instance of conversion or adverse events, suggests this is a safe and feasible method to facilitate VATS resection of solitary lung nodules.

## Figures and Tables

**Table 1:** Clinical characteristics and demographics of two matched groups.

Variables	ENB Group (*n* = 15) Number (%), or Mean ± SD (Range)	CT Group (*n* = 30) Number (%), or Mean ± SD (Range)	*p*-Value
Age, year	54.4 ± 10.9 (38~72)	56.3 ± 11.2 (36~77)	0.547
Male	10 (70.5%)	24 (80.0%)	0.152
Ever smoker	8 (53.3%)	14 (42.1%)	
Height, cm	162.4 ± 7.8(149.0–178.5)	163.0 ± 8.5(150.0–179.0)	0.673
Weight, kg	60.8 ± 7.5 (49.0–76.5)	61.0 ± 8.0 (51.0–78.0)	0.754
FVC (L)	2.9 ± 0.7	2.8 ± 0.6	0.624
FVC (%)	110.8 ± 15.8	108.6 ± 12.1	0.613
FEV1 (L)	2.4 ± 0.6	2.5 ± 0.5	0.364
FEV1 (%)	107.5 ± 21.7	110.5 ± 22.9	0.676
COPD/Emphysematous lung	2 (13.3%)	4 (13.3)	NS
GGO/Solid nodule	14/10	26/22	0.737
Nodule number			
1	6 (40 %)	12 (40.0 %)	NS
2	9 (60 %)	18 (60.0 %)	
Nodule size, cm	1.0 ± 0.5 (0.6–1.5)	1.1 ± 0.6 (0.5–1.8)	0.876
Nodule depth, cm	1.8 ± 0.4 (1.4–2.8)	1.9 ± 0.5 (1.5–2.7)	0.910
Ratio of size/depth	55.8 ± 17.1% (23.8–85.0%)	62.4 ± 15.1% (23.5–90.0%)	0.192
Location			NS
Right	6 (40.0%)	16 (53.3%)	
Left	5 (33.3%)	14 (53.3%)	
Bilateral	4 (26.7%)	0 (0%)	

ENB = electromagnetic navigation bronchoscopy; CT = computed tomography; NS = non-significant; FVC = forced vital capacity; FEV1 = forced expiratory volume in 1 s; COPD = chronic obstructive pulmonary disease; GGO = ground-glass opacity.

**Table 2:** Pathological diagnosis of lung nodules.

Pathological Diagnosis	ENB Group (*n* = 24) Number (%)	CT Group (*n* = 48) Number (%)
Benign lung tumor	3 (12.5%)	5 (10.4%)
Atypical adenomatous hyperplasia	1 (4.2%)	2 (4.2%)
Organizing pneumonia	1 (4.2%)	1 (2.1%)
Intrapulmonary lymph node	1 (4.2%)	2 (4.2%)
Primary lung adenocarcinoma	15 (62.5%)	38 (79.2%)
Other malignancies	6 (25%)	5 (10.4%)
Metastatic colon cancer	4(16.7%)	3 (6.3%)
Metastatic breast cancer	1(4.2%)	1 (2.1%)
Metastatic renal cell carcinoma	0 (0 %)	1 (2.1%)
Metastatic osteosarcoma	1 (4.2%)	0 (0 %)

**Table 3:** Localization and surgery results for the ENB and CT groups.

Variables	ENB Group (*n* = 15) Number (%), or Mean ± SD (Range)	CT Group (*N* = 30) Number (%), or Mean ± SD (Range)	*p*-Value
Global time (min)	143.4 ± 58 (64–205)	258.0 ± 124 (110–580)	0.002
Localization time (min)	21.8 ± 12.5 (8–38)	26.3 ± 14.0 (13–51)	0.299
Surgery time (min)	121.8 ± 41.5 (51–167)	110.7 ± 21.3 (89–142)	0.240
Chest tube drainage (days)	1.33 ± 0.8 (0–2)	1.7 ± 0.8 (1–4)	0.151
Hospital stay (days)	2.33 ± 0.8 (1–4)	2.7 ± 0.8 (1–5)	0.573
Localization complications			
Pneumothorax	1 (6.7%)	11 (36.7%)	0.032
Lung focal hemorrhage	3 (20.0%)	13 (43.3%)	0.226
Diaphragm injury	0 (0%)	1 (3.3%)	NS
Failed to localization	1 (6.7%)	3 (10.0%)	0.711
Conversion to mini-thoracotomy	1 (6.7%)	3 (10.0%)	0.711
Margin involved	0 (0%)	0 (0%)	NS

NS = non-significant.
